# Insulin granule biogenesis and exocytosis

**DOI:** 10.1007/s00018-020-03688-4

**Published:** 2020-11-04

**Authors:** Muhmmad Omar-Hmeadi, Olof Idevall-Hagren

**Affiliations:** Department of Medical Cell Biology, Uppsala University, Biomedical Centre, Husargatan 3, 75123 Uppsala, Sweden

**Keywords:** Diabetes, Lipids, β-Cell, Insulin

## Abstract

Insulin is produced by pancreatic β-cells, and once released to the blood, the hormone stimulates glucose uptake and suppresses glucose production. Defects in both the availability and action of insulin lead to elevated plasma glucose levels and are major hallmarks of type-2 diabetes. Insulin is stored in secretory granules that form at the trans-Golgi network. The granules undergo extensive modifications en route to their release sites at the plasma membrane, including changes in both protein and lipid composition of the granule membrane and lumen. In parallel, the insulin molecules also undergo extensive modifications that render the hormone biologically active. In this review, we summarize current understanding of insulin secretory granule biogenesis, maturation, transport, docking, priming and eventual fusion with the plasma membrane. We discuss how different pools of granules form and how these pools contribute to insulin secretion under different conditions. We also highlight the role of the β-cell in the development of type-2 diabetes and discuss how dysregulation of one or several steps in the insulin granule life cycle may contribute to disease development or progression.

## Introduction

Insulin is the major blood-glucose lowering hormone, and it acts by promoting glucose uptake and storage and by suppressing glucose production. A single cell-type, the β-cell, is solely responsible for all insulin production and secretion. These cells are located within the islets of Langerhans, micro-organs scattered throughout the pancreas, where it is mixed with other endocrine cells involved in blood glucose regulation. Insulin is stored in large, dense-core granules and is released to the circulation in response to elevated plasma glucose concentrations. Insulin secretion is largely controlled at the level of the β-cell, and a single β-cell, taken out of the pancreas, retains the ability to release insulin in response to glucose. This process begins with glucose uptake and metabolism to ATP that subsequently closes ATP-sensitive K^+^-channels, resulting in membrane depolarization, opening of voltage-dependent Ca^2+^ channels, Ca^2+^ influx and insulin granule exocytosis. Insulin granules can be divided into two functionally distinct pools; one containing granules that are release competent and one containing granules that are not. The release competent granules are found immediately adjacent to the plasma membrane and their release gives rise to a sharp increase in plasma insulin known as the first phase of secretion. This granule pool is gradually depleted during prolonged stimulation, and new granules need to be recruited to sustain insulin secretion and maintain blood glucose homeostasis. This process involves both mobilization of granules from the larger reserve pool and de novo generation of insulin granules. Failure to appropriately secrete insulin results in impaired blood glucose control and is a hallmark of type-2 diabetes (T2D). In this review, we describe the molecular steps controlling granule formation at the Golgi and the subsequent granule maturation, transport, docking to and fusion with the plasma membrane. We highlight the role of lipids in multiple regulatory steps in insulin granule biogenesis and release and also discuss how defects in granule biogenesis and release contribute to diabetes development.

## Preproinsulin synthesis

The mammalian insulin gene encodes a single chain precursor protein, preproinsulin, which matures into active insulin through a series of proteolytic reactions. The β-cell-specific expression of insulin is achieved by a glucose-dependent transcriptional program [1]. Post-transcriptional mechanisms, such as stabilization of insulin mRNA by mRNA-binding proteins, also contribute to glucose-effects on insulin production [2], as does translational mechanisms, including stimulation of translation initiation and elongation [3]. As preproinsulin mRNA is translated, the N-terminal signal peptide is recognized by signal recognition particles that direct the ribosome to the ER and facilitate preproinsulin translocation across the ER membrane [3, 4], where the signal peptide is removed [5, 6]. The resulting proinsulin molecules fold with the help of chaperones, including Glucose-regulated protein 94 (GRP94) [7], and form stabile hexamers through interactions with zinc ions [8]. After passing the quality control checkpoint, proinsulin is transported to the cis-face of the Golgi apparatus via the ER-Golgi interface compartment. Following modifications by Golgi-resident enzymes, the proinsulin molecules reach the trans-Golgi network (TGN).

## *trans-*Golgi network sorting of proinsulin and formation of immature secretory granules

Insulin secretory granule (ISG) cargo, including proinsulin, is packaged into nascent granules that bud off from the TGN. Consensus regarding how sorting of ISG cargo is achieved is lacking, and the topic has been extensively debated over the last 30 years. The disagreement concerns whether sorting of proinsulin and other granule components occurs by sequestration in the TGN or by retention in the ISG. There is experimental evidence to support both modes of sorting, and perhaps they are not mutually exclusive but operate in parallel. Irrespective of model, the outcome is the formation of ISG with a composition that differs from that of the TGN.

**Fig. 1:**
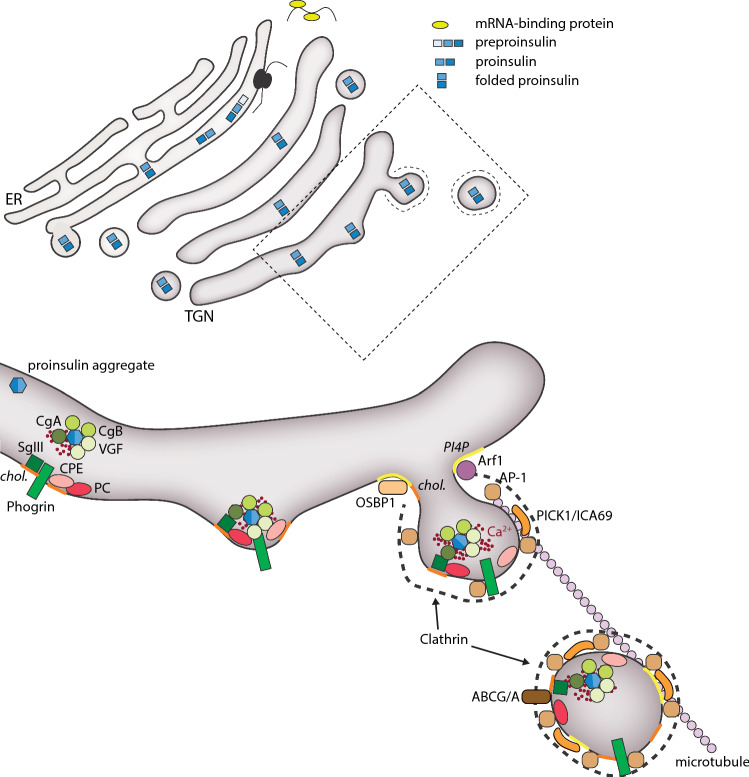
Insulin granule formation at the *trans*-Golgi network. Proinsulin is sequestered to the secretory granule budding site through interactions with proteins like Chromogranin A/B (CgA/B) and VGF, and together these form large aggregates. Additional sorting is provided by membrane-bound proteins like Phogrin and Secretogranin 3 (SgIII), and this occurs in parallel with sorting of proinsulin-processing proteins (PC1/3, PC2 and CPE). Insulin granule budding depends on the action of Arf1 and the membrane bud is stabilized by clathrin, the clathrin-adaptor AP-1, and BAR-domain proteins (PICK1 and ICA69) and is facilitated by high concentrations of the phospholipid PI4P

Soluble proteins destined for ISG can form large aggregates in the presence of millimolar Ca^2+^ and weakly acidic pH [9, 10]. Such conditions exist both in the TGN and ISG, and these aggregates facilitate the condensation of bioactive peptides, such as proinsulin [9]. There is general consensus that aggregation of proinsulin occurs and that this aggregation is important for hormone maturation. However, it is not clear if this condensation occurs in the TGN or in the immature ISG [11]. Numerous proteins of importance for proinsulin condensation have been identified, including chromogranin A (CHGA), chromogranin B (CHGB) and VGF. Mice lacking CHGB exhibit reduced glucose-stimulated insulin secretion, and similar defects are seen in islets and clonal β-cells following transient knockdown of CHGB [12, 13]. Mechanistically, loss of CHGB does not affect ISG biogenesis but it impairs proinsulin processing, leading to reduced insulin content [12]. In contrast, transient knockdown of CHGB impairs ISG biogenesis in clonal β-cells [13]. This difference is likely due to compensatory upregulation of other members of the granin family [14]. β-Cells lacking VGF also exhibit defect proinsulin processing and ISG biogenesis [15]. Noteworthy, the overexpression of a single aggregate-inducing protein is sufficient to induce formation of aggregate-containing vesicles in cells lacking a regulated secretory pathway [16], indicating that at least the aggregating capacity of these proteins is functionally redundant. Such redundancy may also help to explain variable outcomes in loss of function studies. Sorting of ISG cargo also occurs independent of aggregation through interactions with the TGN membrane. This involves both interactions with trans-membrane sorting receptors, such as phogrin (IA-2/2β) [17–21], and with lipids in the TGN or ISG membranes. Whereas sorting of proinsulin to the ISG largely depends on aggregation, sorting of the enzymes involved in its processing to biologically active insulin, including carboxypeptidase E (CPE) and the prohormone convertases PC1/3 and PC2, depends on membrane interactions [22–24]. How sorting through membrane interaction occurs is not fully understood, but it seems to depend on the high cholesterol content (50–70 mol%) of the TGN and ISG membrane [25, 26]. Most ISG cholesterol likely originates from the TGN membrane and depend on directed cholesterol delivery via oxysterol-binding protein 1 (OSBP-1) and members of the ATP-binding cassette (ABC) cholesterol transporters. Indeed, loss of function experiments has confirmed roles of these proteins in ISG biogenesis [25–28]. The TGN membrane is also rich in phosphatidylinositol 4-phosphate (PI4P), a lipid that is required for the formation of ISGs [29–31]. PI4P, together with active, GTP-bound Arf1, recruits AP-1 and other coat proteins [32], resulting in the assembly of a clathrin coat around the budding ISG [33]. Diacylglycerol (DAG) is another lipid that accumulates in the TGN, where it generates membrane curvature that facilitates ISG budding [34, 35] and promotes fission through activation of PKD [36]. The fission process also depends on Golgi-derived microtubules which provide a scaffold for the budding ISG [37] (Fig. 1).

In summary, the formation of ISG at the TGN depends on the coordinated activity of membrane-localized and soluble proteins and their interactions with specific membrane lipids. Surprisingly little research has focused on elucidating the early steps of ISG biogenesis, and most of it has focused on how insulin and its processing enzymes are delivered to the granules. Information regarding to what extent other proteins key to ISG maturation and release are enriched on the granules already at the site of formation is largely missing.

## Insulin secretory granule maturation

The TGN plays an important role in defining the content of ISG, but the sorting process also continues through clathrin-mediated membrane retrieval after granule formation [38–40]. The clathrin-mediated sorting at the ISG is thought to be important for removal of proteolytic enzymes and proteins destined for other organelles, and results in a reduction in ISG size [41, 42]. The ISG additionally undergo a series of maturation steps, where acidification and removal of coat proteins coincide with the conversion of proinsulin to insulin [39, 40, 43]. The entire maturation process, estimated from pulse-chase experiments of radio-labelled proinsulin, takes around 3 h [44]. This is much shorter than the half-life of insulin, which is around 3 days [45], consistent with most insulin being stored in a reserve pool of granules.

The ISG lumen is acidified in a glucose-dependent manner [46] through the action of the vesicular ATP-dependent proton pump (V-ATPase), which in turn increases the activity of the proinsulin converting enzymes PC1/3 and PC2 [39, 44, 47]. These enzymes cleave off a fragment (C-peptide) from proinsulin, converting it into insulin. Both C-peptide and insulin are subsequently used as substrates by CPE, which removes basic residues at the respective cleavage site [48]. The acidification starts shortly after budding from the TGN and occurs over a time-period of around 30 min, during which proinsulin is converted to insulin and the clathrin coat is lost [43, 49]. The mechanism that controls uncoating of ISG is relatively poorly characterized, at least in part because this pool of clathrin-coated vesicles is difficult to separate from clathrin-coated endocytic vesicles of plasma membrane origin. One elegant study followed the lifetime of TGN-derived clathrin-coated vesicles in kidney epithelial cells by rapid 3D imaging and found that these vesicles had a lifetime of around 40 s [50]. This is in sharp contrast to the estimated lifetime of the clathrin-coated ISG, but similar to the lifetime of an endocytic clathrin-coated vesicle [51]. It is possible that the lifetime of clathrin-coated ISG in β-cells is longer, but it still puts into question the importance of clathrin uncoating for the correct maturation of insulin [41, 52]. Mechanistically, the uncoating of ISG likely involves the adaptor protein Auxilin and the cytosolic heat shock cognate protein complex 70 (Hsc70) [53]. Auxilin is recruited to the clathrin-coated granules, likely via binding to phosphoinositides, and subsequently recruits Hsc70, which initiates ATP-dependent removal of clathrin and other coat proteins [53–55]. The clathrin coat stabilizes the ISG, but this function is also shared with other proteins, including the BAR domain proteins PICK1 and ICA69 [56]. Loss of either of these proteins results in impaired proinsulin processing and in reduced synthesis of mature insulin, possibly trough impaired sorting of PC1/3 to the ISG [32, 57].

Insulin is stored as a hexamer inside the uncoated ISG through the interaction with calcium and zinc ions. Part of the Zn^2+^ comes from the Golgi and ER [58], but ISG are also equipped with Zn^2+^ transporters, where ZnT8 is particularly well-studied, since mutations in its gene, SLC30A8, are associated with increased susceptibility to T2D [59]. Loss of function studies have revealed a role of ZnT8 in the regulation of proinsulin processing and ISG biogenesis, but the phenotypes are typically mild and its importance for glucose homeostasis is not firmly established [60]. In fact, recent work suggests that loss of function mutations in ZnT8 might even protect against T2D [61]. Interestingly, the high-risk mutation in ZnT8 is a gain-of-function mutation that increases Zn^2+^ transport [62]. These observations suggest that reducing the activity of ZnT8 might be considered a future therapeutic approach to the treatment of T2D, although care must be taken, since massive impairment of Zn^2+^ uptake strongly inhibits insulin production and secretion [63].

**Fig. 2:**
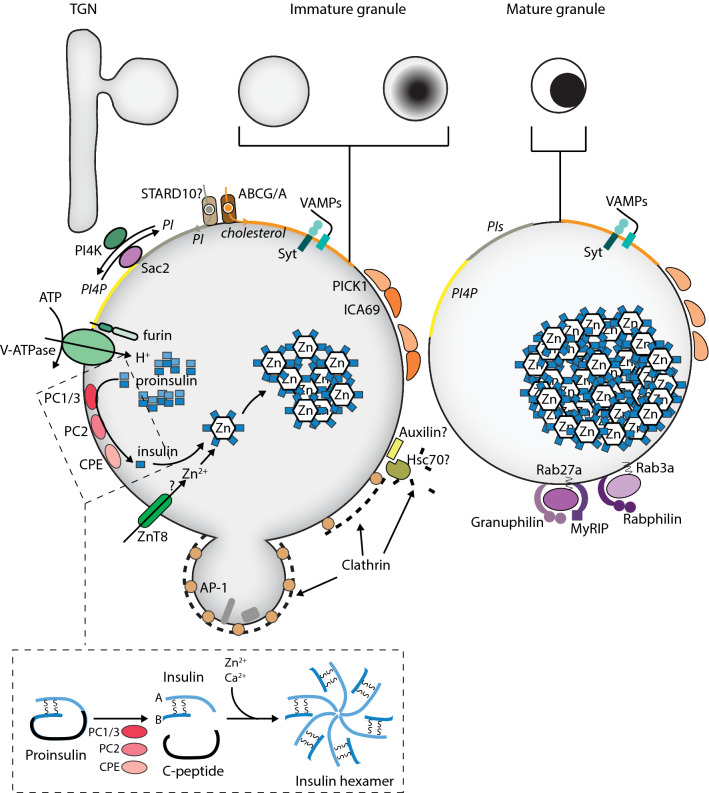
Insulin granule maturation. Proinsulin processing to insulin occurs inside the granule. The proteolytic conversion of insulin requires granule acidification by the H^+^-transporting V-ATPase, which in turn activates the prohormone-processing enzymes (PC1/3, PC2 and CPE). Insulin is stored as a hexamer in complex with Zn^2+^, and at least some of the Zn^2+^ accumulation occurs via ZnT8-mediated transport. Granule-localized cholesterol transporters (ABCG/A) and phosphoinoside-transporting (STARD10) and modulating (PI4K, Sac2) proteins are responsible for changes in the lipid composition of the granule. During maturation, the granule shrinks due to clathrin-dependent retrieval of membrane and cargo for sorting or degradation. Maturation also involves removal of the clathrin coat, which likely depends on the action of Auxilin and Hsc70, and acquisition of factors required for granule transport and docking, such as Rab27a and Rab3 and their corresponding effector proteins (Granuphilin and Rabphilin)

Lipids play important roles in ISG maturation. The cholesterol content of insulin granules is high, at least in part through the action of granule-localized ABC cholesterol transporters [64, 65]. β-Cells lacking the ABCG1/ABCA12 cholesterol transporters or treated with cholesterol synthesis inhibitors present with enlarged ISG and exhibit reduced glucose-stimulated insulin secretion [64–66]. The mechanism of cholesterol action is not clear, but it has been shown that cholesterol transport is needed to protect newly formed ISG against lysosomal degradation [28]. However, excess cholesterol also impairs β-cell function, causing ISG enlargement and impaired functionality by interfering with the localization of exocytic proteins to the ISG [67]. It seems that maintaining cholesterol levels within a narrow range is required for normal ISG biogenesis in β-cells. Indeed, alterations in cholesterol homeostasis have been described in T2D models [64]. In addition to cholesterol and its transporting proteins, steroidogenic acute regulatory protein-related lipid transfer protein 10 (STARD10), which transports phospholipids, has also been implicated in ISG biogenesis [68]. High risk alleles for T2D have been mapped to the *stard10* genomic locus, and risk allele carriers present with reduced STARD10 mRNA levels. Moreover, β-cell-specific loss of STARD10 results in impaired glucose-stimulated insulin secretion [68]. It appears that STARD10 affects insulin secretion both at the level of ISG biogenesis and at more distal steps, but without knowledge of its cellular localization and lipid preference it is difficult to understand its role in β-cells. Phospholipids have also been more directly implicated in ISG maturation. The levels of PI4P are high on the ISG surface, at least in part via the presence of ISG-localized PI4-kinases [69], but the lipid somehow also needs to be removed by the action of the 4′-phosphates Sac2 prior to ISG docking at the plasma membrane [70]. Such dynamic changes in the concentration of a phosphoinositide are reminiscent of the well-characterized phosphoinositide cascades that drive membrane and cargo trafficking in the endo-lysosomal compartment [71], and may indicate that there are similarities between the mechanisms that control cargo uptake and release in secretory cells [72]. Mass spectrometric analysis has revealed dramatic glucose-induced changes in the phosphoinositide composition of ISG; however, it is not known which lipid species that are affected [73] (Fig. 2).

## Insulin secretory granule transport

**Fig. 3:**
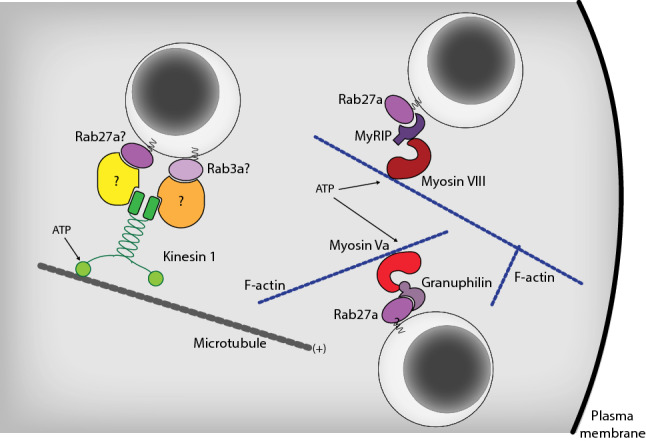
Insulin granule transportation. Long-range transport of insulin granules occurs along microtubules and depend on the anterograde motor protein Kinesin-1. Short-range transport close to the plasma membrane instead occurs along F-actin tracks and depends on interactions between mature insulin granule proteins and the motor proteins Myosin Va and Myosin VIII

Acute stimulation of insulin secretion primarily involves the release of granules already present at the plasma membrane. Prolonged stimulation requires mobilization of granules from the reserve pool through a mechanism that depends on glucose and a granule transport machinery [74]. There are two main transport routes in cells; actin filaments and microtubules. Filamentous actin (F-actin) is dynamically regulated by glucose and influence insulin secretion at numerous stages, including granule transport to the plasma membrane [75, 76]. Myosin Va is an F-actin-dependent transport protein that binds ISG through interactions with granuphilin, Rab27a and Rabphilin [76–78]. Rab27a also binds to Exophilin-8/MyRIP, which in turn anchors ISG to the cortical F-actin network via interactions with Myosin VII, thereby stabilizing granules at the plasma membrane [79]. F-actin filaments are important for short-range movement of ISG close to the plasma membrane, whereas long-range movements instead involve kinesin-dependent microtubule transport [80]. ATP derived from glucose metabolism promotes microtubule-dependent movement of ISG, and interference with kinesin-1 function results in the selective suppression of sustained insulin secretion [80–82]. Interestingly, microtubule-dependent ISG transport primarily involves newly synthesized ISG, indicating that the granule membrane composition is altered with ageing to prevent binding to microtubules [83]. Kinesin-1 attaches to ISG via an adaptor protein, and it is, therefore, easy to envisage how changes in granule membrane composition can affect granule mobility. The identity of the adaptors and cargo proteins involved in microtubule-dependent transport are not known, although both Rab3a and Rab27a have been shown to be involved in axonal transport of synaptic vesicle precursors in neurons [84]. Recently, it has been proposed that microtubules may also play a negative role in insulin secretion regulation by retrograde transport of ISG from the plasma membrane [85]. The regulation of ISG transport is clearly complicated, and the relative importance of F-actin, microtubules and random diffusion for maintaining appropriate rates of insulin secretion is still not understood. Determining the mechanisms that control insulin granule attachment to motor proteins and investigating the possibility that the mobility of different granules pools is governed by different transport mechanisms could help to answer these questions (Fig. 3).

## Dynamics of insulin secretion

**Fig. 4:**
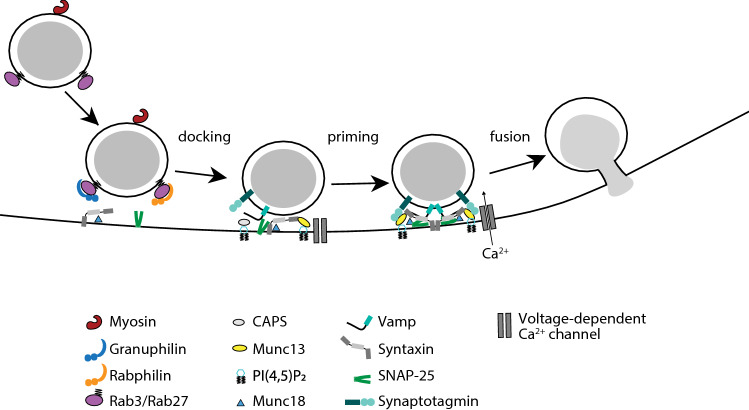
Insulin granule exocytosis. The three main stages of insulin granule exocytosis at the plasma membrane, docking, priming, and fusion are regulated by interactions between specific sets of proteins and lipids (see text for details)

Numerous dietary components, such as glucose, proteins and fatty acids, can stimulate insulin secretion. Glucose is nevertheless considered the major physiological stimulus for insulin secretion, and the other components mostly act through amplification of the glucose-triggered secretory response [86]. Insulin secretion in response to a step increase in glucose concentration occurs in a biphasic manner composed of a transient first phase, lasting only a few minutes, followed by a prolonged, sustained second phase built up of regular pulses. This release kinetics has been observed at different organizational levels, from the portal vein of the perfused rat pancreas to individual β-cells [87–90], indicating that β-cell-intrinsic mechanisms are responsible for the biphasic secretory response. A common explanation is that exocytosis of different functional granule pools gives rise to the two phases. Morphologically, electron microscopy has shown that initial stimulation of insulin secretion selectively depletes ISG that are in the vicinity of the plasma membrane [91]. The granules undergoing fusion are classified into the readily releasable pool (RRP), which constitute a subset of plasma membrane-docked granules that are primed with a fully assembled exocytosis machinery. The vast majority (98%) of ISG belongs to a reserve pool located deeper within the β-cell, and release of these granules requires trafficking and recruitment to the same release sites. Consequently, the second phase of insulin secretion is much slower and long-lasting [91–95]. Electrophysiological characterization has confirmed the presence of two populations of granules with different release kinetics; a small population of around 100 granules that are rapidly released (RRP), and a larger population with a slower release rate [95]. Similar conclusions were also drawn from modelling studies [96]. In light of the above-mentioned findings, the prevailing hypothesis is that the RRP is largely responsible for the first phase of secretion, while sustained secretion relies on recruitment of granules from the reserve pool [93, 97]. Studies addressing the molecular mechanisms of biphasic insulin secretion have relied heavily on the use of glucose or non-physiological secretagogues. Importantly, biphasic insulin secretion is also observed in vivo in humans after oral intake of a mixed meal (glucose, protein and fat), demonstrating that the biphasic release kinetics is physiological [98]. The first phase of insulin secretion plays an important role in postprandial glucose homeostasis, and it is often lost or reduced in early stages of type-2 diabetes (T2D; see later sections) [99] (Fig. 4).

## Release probability is determined at the level of the insulin secretory granule

The morphologically visible separation of ISG into a docked and undocked pool suggests that β-cells have mechanisms for targeting granules to the plasma membrane. The challenge is to understand the molecular basis for these functionally defined granule pools. Live-cell imaging during phasic insulin secretion has resulted in a model, where the ISG are not distinguished based on their physical location per se but by their modes of exocytosis [96, 97, 99, 100]. Primary exocytosis occurs through a process involving ISG docking to the plasma membrane, followed by priming and Ca^2+^-dependent release. This is generally believed to be the most important mode of ISG release. In newcomer granule exocytosis, ISG instead immediately fuse with the plasma membrane with either minimal or no residence time at the plasma membrane [99]. Moreover, kiss-and-run exocytosis, where ISG briefly contact the plasma membrane through a transient fusion pore, has also been described in β-cells [101]. ISG can also undergo compound exocytosis, involving the simultaneous release of several granules that had fused with each other inside the cytoplasm [102].

The SNARE model of exocytosis dictates that the t-SNARE proteins SNAP-25 and Syntaxin in the plasma membrane interact with the v-SNARE protein VAMP on the ISG membrane to form a SNARE complex. Exocytosis also depends on accessory proteins, including Munc18, Munc13, Rabs and active zone proteins, such as RIMs, Piccolo and Bassoon (reviewed in [74]). Differences in SNARE complex composition determines the mode of exocytosis. Primary exocytosis is mediated by VAMP2, syntaxin1A, and SNAP-25, and depends on Munc18-1, while newcomer granule exocytosis involves Munc18b/c activation of syntaxin3/4, SNAP-25 and VAMP8 [103–107]. The importance of the different modes of exocytosis for normal β-cell function is not clear, but they may contribute to the strength and plasticity of insulin secretion.

## Insulin secretory granule docking

Docked granules are physically immobilized at specialized docking sites at the plasma membrane [95, 108–110]. Several molecular interactions have been shown to play essential roles in docking-site formation. Small GTPases of the Rab family allow ISG to tether at the correct target membrane. The granules that successfully tether to their release sites are equipped with Rab3, which interacts with Rab3-interacting molecule RIM2α at the plasma membrane [111]. This is followed by the clustering of the SNARE protein syntaxin1 and its binding partner munc18-1, which together initiate and orchestrate the building of the docking site at the plasma membrane [112]. Munc18-1 binds syntaxin1 in a dormant “closed” conformation, where the SNARE domain is not accessible, thus preventing early recruitment of other SNARE proteins [113–116]. Both syntaxin1 and munc18-1 are required for stabile ISG docking at the plasma membrane [117, 118]. Syntaxin clustering also depends on cholesterol [119] and other membrane lipids [120]. In particular, PI(4,5)P_2_ has been proposed to have a role in organizing the plasma membrane by acting, together with SNARE proteins, as beacons for incoming granules [121, 122]. Indeed, both syntaxin-1 and PI(4,5)P_2_ have been shown to form microdomains in cell-free membranes and fixated cells that at least partially colocalize with docked granules [123–125]. However, their presence in the plasma membrane of living cells is controversial and may depend on cell type and experimental methods [126–128]. However, selective removal of PI(4,5)P_2_ at docking sites affects tethering and docking [129].

## Insulin secretory granule priming and release

Docking is required, but not sufficient, for exocytosis. Morphological analyses indicate that a few hundred granules are docked at the plasma membrane. However, most of these granules are not released upon stimulation but must undergo a maturation process known as priming. The pool of docked granules is likely the main depot for granule priming and replenishment of the RRP [130]. Priming transforms the granules to a state of readiness for fusion, and involves all the molecular rearrangements and ATP-dependent protein and lipid modifications that take place after docking but before fusion. However, the key reactions defining priming are not fully elucidated. Priming is thought to involve conformational changes and partial formation of the core SNARE complex [131, 132]. Munc13 and Ca^2+^-dependent activator protein in secretion (CAPS) are thought to ensure proper assembly of the SNARE complex [133], and to attract Ca^2+^ channels to the release site [134]. They catalyse the transition of closed syntaxin‐1 into its open conformation, leading to partially zippered SNARE complexes involving SNAP-25 and VAMP2 [135]. The docking factor Munc18-1 is also required for priming by facilitating the Munc13-dependent SNARE assembly and serving as a template that enables syntaxin-1 and VAMP2 interactions [135, 136]. Munc13, together with Munc18, also prevent NSF‐dependent disassembly of the docking complex [133]. CAPS-1/2 are also essential components of the priming machinery, and they contain a sequence stretch with homology to the priming domain of Munc13 [137]. The partially formed SNARE complex is stabilized by complexin [138] and tomosyns [139] that prevent spontaneous exocytosis. Upon Ca^2+^ influx, ISG undergo exocytosis in a manner dependent on Synaptotagmin and Doc2B. Both proteins are equipped with Ca^2+^-binding C2 domains which also exhibit distinct binding properties for SNARE proteins, Munc18 and phospholipids in the plasma membrane [140, 141]. Activation of these Ca^2+^ sensors releases the exocytic clamp and enable full SNARE zippering and granule fusion with the plasma membrane.

Lipids, in particular PI(4,5)P_2_, plays important roles during granule priming and release. For example, the ATP requirement for priming involves PI(4,5)P_2_ synthesis [142–144]. It has also been shown that the number of primed granules and the rate of sustained secretion relates to PI(4,5)P_2_ levels [69, 127, 145]. This is likely due to PI(4,5)P_2_-dependent recruitment and activation of cytosolic proteins, e.g., CAPS and Munc13, at specific locations on the plasma membrane [137, 146–148]. It is worth to mention that β-cells are polarized within islets, and ISG exocytosis is directed towards the vasculature. These cellular domains are enriched in proteins involved in cell adhesion (F-actin, E-cadherin and integrins) [149] and associated with neuronal presynaptic proteins (RIM2α, ELKS, Liprin, and Piccolo) [150]. In isolated cells, polarized secretion is maintained to some extent. Spatial patterning of ISG recruitment and fusion occurs at polarized sites especially when the SNARE clusters also include Ca^2+^ channels [134, 151]*.* Local activation of integrin signalling mimics the vascular face of the cell and contribute to polarized secretion [152, 153]. Further work is required to understand the role of this active zone-like organization in regulating physiological insulin secretion.

## Do abnormalities in the β-cell secretory machinery contribute to type-2 diabetes?

The functional impairment of β-cells in T2D has been the topic of intense investigation for decades. Many factors involved in T2D pathogenesis have been identified, including inflammatory stress, ER stress, metabolic and oxidative stress, amyloid stress, changes in the structural integrity of the islet and defects in the insulin secretory machinery [154]. The relative importance of these different factors is not known and, due to the strong genetic component of T2D, may even vary from one individual to another. In this review we have chosen to focus on the link between T2D and defects in the insulin secretory machinery. There is evidence that defects in the ISG release machinery, together with changes in metabolism and electrical activity, contribute to the functional β-cell decline in T2D. This disease is characterized by the loss of biphasic glucose-stimulated insulin secretion, both in vivo and in isolated islets. Loss of the first phase is one of the earliest manifestations of T2D [155], and might be explained by inadequate β-cell function [156, 157]. β-Cells from T2D organ donors exhibit decreased numbers of docked granules, at least in part due to downregulation of docking factors, such as and Syntaxin-1, Munc18, Munc13, Rim2, Rab3a, and Rabphilin3a [158], and recent advances in single-cell sequencing are likely to expand this list [159]. Moreover, ISG docking in T2D β-cells also loose spatial control and occurs at random locations at the plasma membrane, indicating the absence of a specific docking signal [151]. The reduced docking results in impaired ISG exocytosis in these cells [151, 158]. The ability of antidiabetic drugs, such as sulfonylureas, to restore biphasic insulin secretion indicates that β-cell defects also result from impaired electrical activity [160, 161]. It also highlights the role of the metabolic changes that accompany T2D, since the inability of glucose to fully depolarize the β-cell is the principal reason for the loss of biphasic insulin secretion. T2D also disrupts the architectural organization of individual ISG release sites. For example, the coupling of L-type Ca^2+^-channels to the release site at the plasma membrane is lost in diabetic β-cells, leading to slowed exocytosis that is de-synchronized with Ca^2+^-influx [134]. Altered clustering of Ca^2+^ channels and Syntaxin-1 has also been observed after long-term culture in elevated free fatty acids or glucose [162]. Together with previously discussed alterations ISG biogenesis and maturation in T2D, these observations highlight the central role of the β-cell secretory machinery in T2D development and progression.

## Concluding remarks

Extensive work over the past century has brought about remarkable advance in our understanding of the nature of ISG biogenesis and secretion. It has also been firmly established that defects in ISG biogenesis and release are hallmarks of type-2 diabetes. However, many challenges still remain to fully understand insulin physiology. A major open question is what drives changes in β-cell function and insulin secretion in type-2 diabetes. It is currently not clear to what extent loss of insulin secretion is a primary defect or a consequence of, e.g., metabolic changes. Understanding this relationship will be crucial when devising new strategies to improve or restore β-cell function. Other important questions worth further exploring are the importance of the active zone-like organization for insulin secretion and to what extent, if any, different modes of exocytosis contribute to the pathology of T2D. Further studies are also needed to shed light on insulin granule heterogeneity and turnover to fully understand the dynamics of insulin secretion. Another aspect that deserves further investigation is the role of the local microenvironment in regulating insulin secretion from β-cells, including auto- para- and juxtacrine interactions within the islets and cell polarization and the generation of cellular domains. Such experiments will require studies of β-cells within intact islets, ideally in vivo, something that has only recently been made possible thanks to the development of sophisticated imaging techniques and model systems. These techniques, together with single-cell approaches to identify diabetes genes and powerful genetic techniques for target validation may finally enable complete mapping of the insulin secretory granule pathway, a route where both detours and shortcuts may result in diabetes development or progression.

## References

[1] Andrali SS, Sampley ML, Vanderford NL, Ozcan S (2008). Glucose regulation of insulin gene expression in pancreatic beta-cells. Biochem J.

[2] Tillmar L, Carlsson C, Welsh N (2002). Control of insulin mRNA stability in rat pancreatic islets. Regulatory role of a 3'-untranslated region pyrimidine-rich sequence. J Biol Chem.

[3] Magro MG, Solimena M (2013). Regulation of beta-cell function by RNA-binding proteins. Mol Metab.

[4] Greenman IC, Gomez E, Moore CE, Herbert TP (2005). The selective recruitment of mRNA to the ER and an increase in initiation are important for glucose-stimulated proinsulin synthesis in pancreatic beta-cells. Biochem J.

[5] Dodson G, Steiner D (1998). The role of assembly in insulin's biosynthesis. Curr Opin Struct Biol.

[6] Patzelt C, Labrecque AD, Duguid JR, Carroll RJ, Keim PS, Heinrikson RL, Steiner DF (1978). Detection and kinetic behavior of preproinsulin in pancreatic islets. Proc Natl Acad Sci USA.

[7] Ghiasi SM, Dahlby T, Hede Andersen C, Haataja L, Petersen S, Omar-Hmeadi M, Yang M, Pihl C, Bresson SE, Khilji MS, Klindt K, Cheta O, Perone MJ, Tyrberg B, Prats C, Barg S, Tengholm A, Arvan P, Mandrup-Poulsen T, Marzec MT (2019). Endoplasmic reticulum chaperone glucose-regulated protein 94 is essential for proinsulin handling. Diabetes.

[8] Huang XF, Arvan P (1995). Intracellular transport of proinsulin in pancreatic beta-cells. Structural maturation probed by disulfide accessibility. J Biol Chem.

[9] Gerdes HH, Glombik MM (1999). Signal-mediated sorting to the regulated pathway of protein secretion. Ann Anat.

[10] Kienzle C, von Blume J (2014). Secretory cargo sorting at the trans-Golgi network. Trends Cell Biol.

[11] Arvan P, Halban PA (2004). Sorting ourselves out: seeking consensus on trafficking in the beta-cell. Traffic.

[12] Obermuller S, Calegari F, King A, Lindqvist A, Lundquist I, Salehi A, Francolini M, Rosa P, Rorsman P, Huttner WB, Barg S (2010). Defective secretion of islet hormones in chromogranin-B deficient mice. PLoS ONE.

[13] Bearrows SC, Bauchle CJ, Becker M, Haldeman JM, Swaminathan S, Stephens SB (2019). Chromogranin B regulates early-stage insulin granule trafficking from the Golgi in pancreatic islet beta-cells. J Cell Sci.

[14] Wollam J, Mahata S, Riopel M, Hernandez-Carretero A, Biswas A, Bandyopadhyay GK, Chi NW, Eiden LE, Mahapatra NR, Corti A, Webster NJG, Mahata SK (2017). Chromogranin A regulates vesicle storage and mitochondrial dynamics to influence insulin secretion. Cell Tissue Res.

[15] Stephens SB, Edwards RJ, Sadahiro M, Lin WJ, Jiang C, Salton SR, Newgard CB (2017). The prohormone VGF regulates beta cell function via insulin secretory granule biogenesis. Cell Rep.

[16] Beuret N, Stettler H, Renold A, Rutishauser J, Spiess M (2004). Expression of regulated secretory proteins is sufficient to generate granule-like structures in constitutively secreting cells. J Biol Chem.

[17] Wasmeier C, Bright NA, Hutton JC (2002). The lumenal domain of the integral membrane protein phogrin mediates targeting to secretory granules. Traffic.

[18] Torii S, Saito N, Kawano A, Zhao S, Izumi T, Takeuchi T (2005). Cytoplasmic transport signal is involved in phogrin targeting and localization to secretory granules. Traffic.

[19] Saito N, Takeuchi T, Kawano A, Hosaka M, Hou N, Torii S (2011). Luminal interaction of phogrin with carboxypeptidase E for effective targeting to secretory granules. Traffic.

[20] Kubosaki A, Gross S, Miura J, Saeki K, Zhu M, Nakamura S, Hendriks W, Notkins AL (2004). Targeted disruption of the IA-2beta gene causes glucose intolerance and impairs insulin secretion but does not prevent the development of diabetes in NOD mice. Diabetes.

[21] Henquin JC, Nenquin M, Szollosi A, Kubosaki A, Notkins AL (2008). Insulin secretion in islets from mice with a double knockout for the dense core vesicle proteins islet antigen-2 (IA-2) and IA-2beta. J Endocrinol.

[22] Dhanvantari S, Arnaoutova I, Snell CR, Steinbach PJ, Hammond K, Caputo GA, London E, Loh YP (2002). Carboxypeptidase E, a prohormone sorting receptor, is anchored to secretory granules via a C-terminal transmembrane insertion. Biochemistry.

[23] Arnaoutova I, Smith AM, Coates LC, Sharpe JC, Dhanvantari S, Snell CR, Birch NP, Loh YP (2003). The prohormone processing enzyme PC3 is a lipid raft-associated transmembrane protein. Biochemistry.

[24] Assadi M, Sharpe JC, Snell C, Loh YP (2004). The C-terminus of prohormone convertase 2 is sufficient and necessary for Raft association and sorting to the regulated secretory pathway. Biochemistry.

[25] Dhanvantari S, Loh YP (2000). Lipid raft association of carboxypeptidase E is necessary for its function as a regulated secretory pathway sorting receptor. J Biol Chem.

[26] Hosaka M, Suda M, Sakai Y, Izumi T, Watanabe T, Takeuchi T (2004). Secretogranin III binds to cholesterol in the secretory granule membrane as an adapter for chromogranin A. J Biol Chem.

[27] Kruit JK, Wijesekara N, Fox JE, Dai XQ, Brunham LR, Searle GJ, Morgan GP, Costin AJ, Tang R, Bhattacharjee A, Johnson JD, Light PE, Marsh BJ, Macdonald PE, Verchere CB, Hayden MR (2011). Islet cholesterol accumulation due to loss of ABCA1 leads to impaired exocytosis of insulin granules. Diabetes.

[28] Hussain SS, Harris MT, Kreutzberger AJB, Inouye CM, Doyle CA, Castle AM, Arvan P, Castle JD (2018). Control of insulin granule formation and function by the ABC transporters ABCG1 and ABCA1 and by oxysterol binding protein OSBP. Mol Biol Cell.

[29] Cruz-Garcia D, Ortega-Bellido M, Scarpa M, Villeneuve J, Jovic M, Porzner M, Balla T, Seufferlein T, Malhotra V (2013). Recruitment of arfaptins to the trans-Golgi network by PI(4)P and their involvement in cargo export. EMBO J.

[30] De Matteis MA, Wilson C, D'Angelo G (2013). Phosphatidylinositol-4-phosphate: the Golgi and beyond. BioEssays.

[31] Santiago-Tirado FH, Legesse-Miller A, Schott D, Bretscher A (2011). PI4P and Rab inputs collaborate in myosin-V-dependent transport of secretory compartments in yeast. Dev Cell.

[32] Cao M, Mao Z, Kam C, Xiao N, Cao X, Shen C, Cheng KK, Xu A, Lee KM, Jiang L, Xia J (2013). PICK1 and ICA69 control insulin granule trafficking and their deficiencies lead to impaired glucose tolerance. PLoS Biol.

[33] Dittie AS, Hajibagheri N, Tooze SA (1996). The AP-1 adaptor complex binds to immature secretory granules from PC12 cells, and is regulated by ADP-ribosylation factor. J Cell Biol.

[34] Tanguy E, Carmon O, Wang Q, Jeandel L, Chasserot-Golaz S, Montero-Hadjadje M, Vitale N (2016). Lipids implicated in the journey of a secretory granule: from biogenesis to fusion. J Neurochem.

[35] Asp L, Kartberg F, Fernandez-Rodriguez J, Smedh M, Elsner M, Laporte F, Barcena M, Jansen KA, Valentijn JA, Koster AJ, Bergeron JJ, Nilsson T (2009). Early stages of Golgi vesicle and tubule formation require diacylglycerol. Mol Biol Cell.

[36] Gehart H, Goginashvili A, Beck R, Morvan J, Erbs E, Formentini I, De Matteis MA, Schwab Y, Wieland FT, Ricci R (2012). The BAR domain protein Arfaptin-1 controls secretory granule biogenesis at the trans-Golgi network. Dev Cell.

[37] Trogden KP, Zhu X, Lee JS, Wright CVE, Gu G, Kaverina I (2019). Regulation of glucose-dependent golgi-derived microtubules by cAMP/EPAC2 promotes secretory vesicle biogenesis in pancreatic beta cells. Curr Biol.

[38] Orci L, Ravazzola M, Amherdt M, Louvard D, Perrelet A (1985). Clathrin-immunoreactive sites in the Golgi apparatus are concentrated at the trans pole in polypeptide hormone-secreting cells. Proc Natl Acad Sci USA.

[39] Orci L, Ravazzola M, Amherdt M, Madsen O, Perrelet A, Vassalli JD, Anderson RG (1986). Conversion of proinsulin to insulin occurs coordinately with acidification of maturing secretory vesicles. J Cell Biol.

[40] Orci L, Ravazzola M, Storch MJ, Anderson RG, Vassalli JD, Perrelet A (1987). Proteolytic maturation of insulin is a post-Golgi event which occurs in acidifying clathrin-coated secretory vesicles. Cell.

[41] Molinete M, Dupuis S, Brodsky FM, Halban PA (2001). Role of clathrin in the regulated secretory pathway of pancreatic beta-cells. J Cell Sci.

[42] Klumperman J, Kuliawat R, Griffith JM, Geuze HJ, Arvan P (1998). Mannose 6-phosphate receptors are sorted from immature secretory granules via adaptor protein AP-1, clathrin, and syntaxin 6-positive vesicles. J Cell Biol.

[43] Orci L, Ravazzola M, Amherdt M, Madsen O, Vassalli JD, Perrelet A (1985). Direct identification of prohormone conversion site in insulin-secreting cells. Cell.

[44] Davidson HW, Rhodes CJ, Hutton JC (1988). Intraorganellar calcium and pH control proinsulin cleavage in the pancreatic beta cell via two distinct site-specific endopeptidases. Nature.

[45] Marsh BJ, Soden C, Alarcon C, Wicksteed BL, Yaekura K, Costin AJ, Morgan GP, Rhodes CJ (2007). Regulated autophagy controls hormone content in secretory-deficient pancreatic endocrine beta-cells. Mol Endocrinol.

[46] Stiernet P, Guiot Y, Gilon P, Henquin JC (2006). Glucose acutely decreases pH of secretory granules in mouse pancreatic islets. Mechanisms and influence on insulin secretion. J Biol Chem.

[47] Rhodes CJ, Lucas CA, Mutkoski RL, Orci L, Halban PA (1987). Stimulation by ATP of proinsulin to insulin conversion in isolated rat pancreatic islet secretory granules. Association with the ATP-dependent proton pump. J Biol Chem.

[48] Davidson HW, Hutton JC (1987). The insulin-secretory-granule carboxypeptidase H. Purification and demonstration of involvement in proinsulin processing. Biochem J.

[49] Steiner DF, Michael J, Houghten R, Mathieu M, Gardner PR, Ravazzola M, Orci L (1987). Use of a synthetic peptide antigen to generate antisera reactive with a proteolytic processing site in native human proinsulin: demonstration of cleavage within clathrin-coated (pro)secretory vesicles. Proc Natl Acad Sci USA.

[50] Kural C, Tacheva-Grigorova SK, Boulant S, Cocucci E, Baust T, Duarte D, Kirchhausen T (2012). Dynamics of intracellular clathrin/AP1- and clathrin/AP3-containing carriers. Cell Rep.

[51] Taylor MJ, Perrais D, Merrifield CJ (2011). A high precision survey of the molecular dynamics of mammalian clathrin-mediated endocytosis. PLoS Biol.

[52] Sahu BS, Manna PT, Edgar JR, Antrobus R, Mahata SK, Bartolomucci A, Borner GHH, Robinson MS (2017). Role of clathrin in dense core vesicle biogenesis. Mol Biol Cell.

[53] Kirchhausen T, Owen D, Harrison SC (2014). Molecular structure, function, and dynamics of clathrin-mediated membrane traffic. Cold Spring Harbor Perspect Biol.

[54] Rothman JE, Schmid SL (1986). Enzymatic recycling of clathrin from coated vesicles. Cell.

[55] He K, Song E, Upadhyayula S, Dang S, Gaudin R, Skillern W, Bu K, Capraro BR, Rapoport I, Kusters I, Ma M, Kirchhausen T (2020). Dynamics of Auxilin 1 and GAK in clathrin-mediated traffic. J Cell Biol.

[56] Herlo R, Lund VK, Lycas MD, Jansen AM, Khelashvili G, Andersen RC, Bhatia V, Pedersen TS, Albornoz PBC, Johner N, Ammendrup-Johnsen I, Christensen NR, Erlendsson S, Stoklund M, Larsen JB, Weinstein H, Kjaerulff O, Stamou D, Gether U, Madsen KL (2018). An amphipathic helix directs cellular membrane curvature sensing and function of the BAR domain protein PICK1. Cell Rep.

[57] Li J, Mao Z, Huang J, Xia J (2018). PICK1 is essential for insulin production and the maintenance of glucose homeostasis. Mol Biol Cell.

[58] Huang L, Yan M, Kirschke CP (2010). Over-expression of ZnT7 increases insulin synthesis and secretion in pancreatic beta-cells by promoting insulin gene transcription. Exp Cell Res.

[59] Sladek R, Rocheleau G, Rung J, Dina C, Shen L, Serre D, Boutin P, Vincent D, Belisle A, Hadjadj S, Balkau B, Heude B, Charpentier G, Hudson TJ, Montpetit A, Pshezhetsky AV, Prentki M, Posner BI, Balding DJ, Meyre D, Polychronakos C, Froguel P (2007). A genome-wide association study identifies novel risk loci for type 2 diabetes. Nature.

[60] Davidson HW, Wenzlau JM, O'Brien RM (2014). Zinc transporter 8 (ZnT8) and beta cell function. Trends Endocrinol Metab.

[61] Dwivedi OP, Lehtovirta M, Hastoy B, Chandra V, Krentz NAJ, Kleiner S, Jain D, Richard AM, Abaitua F, Beer NL, Grotz A, Prasad RB, Hansson O, Ahlqvist E, Krus U, Artner I, Suoranta A, Gomez D, Baras A, Champon B, Payne AJ, Moralli D, Thomsen SK, Kramer P, Spiliotis I, Ramracheya R, Chabosseau P, Theodoulou A, Cheung R, van de Bunt M, Flannick J, Trombetta M, Bonora E, Wolheim CB, Sarelin L, Bonadonna RC, Rorsman P, Davies B, Brosnan J, McCarthy MI, Otonkoski T, Lagerstedt JO, Rutter GA, Gromada J, Gloyn AL, Tuomi T, Groop L (2019). Loss of ZnT8 function protects against diabetes by enhanced insulin secretion. Nat Genet.

[62] Merriman C, Huang Q, Rutter GA, Fu D (2016). Lipid-tuned zinc transport activity of human ZnT8 protein correlates with risk for type-2 diabetes. J Biol Chem.

[63] Syring KE, Boortz KA, Oeser JK, Ustione A, Platt KA, Shadoan MK, McGuinness OP, Piston DW, Powell DR, O'Brien RM (2016). Combined deletion of Slc30a7 and Slc30a8 unmasks a critical role for ZnT8 in glucose-stimulated insulin secretion. Endocrinology.

[64] Sturek JM, Castle JD, Trace AP, Page LC, Castle AM, Evans-Molina C, Parks JS, Mirmira RG, Hedrick CC (2010). An intracellular role for ABCG1-mediated cholesterol transport in the regulated secretory pathway of mouse pancreatic beta cells. J Clin Investig.

[65] Ursino GM, Fu Y, Cottle DL, Mukhamedova N, Jones LK, Low H, Tham MS, Gan WJ, Mellett NA, Das PP, Weir JM, Ditiatkovski M, Fynch S, Thorn P, Thomas HE, Meikle PJ, Parkington HC, Smyth IM, Sviridov D (2020). ABCA12 regulates insulin secretion from beta-cells. EMBO Rep.

[66] Tsuchiya M, Hosaka M, Moriguchi T, Zhang S, Suda M, Yokota-Hashimoto H, Shinozuka K, Takeuchi T (2010). Cholesterol biosynthesis pathway intermediates and inhibitors regulate glucose-stimulated insulin secretion and secretory granule formation in pancreatic beta-cells. Endocrinology.

[67] Bogan JS, Xu Y, Hao M (2012). Cholesterol accumulation increases insulin granule size and impairs membrane trafficking. Traffic.

[68] Carrat GR, Hu M, Nguyen-Tu MS, Chabosseau P, Gaulton KJ, van de Bunt M, Siddiq A, Falchi M, Thurner M, Canouil M, Pattou F, Leclerc I, Pullen TJ, Cane MC, Prabhala P, Greenwald W, Schulte A, Marchetti P, Ibberson M, MacDonald PE, Manning Fox JE, Gloyn AL, Froguel P, Solimena M, McCarthy MI, Rutter GA (2017). Decreased STARD10 expression is associated with defective insulin secretion in humans and mice. Am J Hum Genet.

[69] Olsen HL, Hoy M, Zhang W, Bertorello AM, Bokvist K, Capito K, Efanov AM, Meister B, Thams P, Yang SN, Rorsman P, Berggren PO, Gromada J (2003). Phosphatidylinositol 4-kinase serves as a metabolic sensor and regulates priming of secretory granules in pancreatic beta cells. Proc Natl Acad Sci USA.

[70] Nguyen PM, Gandasi NR, Xie B, Sugahara S, Xu Y, Idevall-Hagren O (2019). The PI(4)P phosphatase Sac2 controls insulin granule docking and release. J Cell Biol.

[71] Di Paolo G, De Camilli P (2006). Phosphoinositides in cell regulation and membrane dynamics. Nature.

[72] Ma CI, Yang Y, Kim T, Chen CH, Polevoy G, Vissa M, Burgess J, Brill JA (2020). An early endosome-derived retrograde trafficking pathway promotes secretory granule maturation. J Cell Biol.

[73] MacDonald MJ, Ade L, Ntambi JM, Ansari IU, Stoker SW (2015). Characterization of phospholipids in insulin secretory granules and mitochondria in pancreatic beta cells and their changes with glucose stimulation. J Biol Chem.

[74] Rorsman P, Ashcroft FM (2018). Pancreatic beta-cell electrical activity and insulin secretion: of mice and men. Physiol Rev.

[75] Arous C, Halban PA (2015). The skeleton in the closet: actin cytoskeletal remodeling in beta-cell function. Am J Physiol Endocrinol Metab.

[76] Varadi A, Tsuboi T, Rutter GA (2005). Myosin Va transports dense core secretory vesicles in pancreatic MIN6 beta-cells. Mol Biol Cell.

[77] Brozzi F, Diraison F, Lajus S, Rajatileka S, Philips T, Regazzi R, Fukuda M, Verkade P, Molnar E, Varadi A (2012). Molecular mechanism of myosin Va recruitment to dense core secretory granules. Traffic.

[78] Ivarsson R, Jing X, Waselle L, Regazzi R, Renstrom E (2005). Myosin 5a controls insulin granule recruitment during late-phase secretion. Traffic.

[79] Fan F, Matsunaga K, Wang H, Ishizaki R, Kobayashi E, Kiyonari H, Mukumoto Y, Okunishi K, Izumi T (2017). Exophilin-8 assembles secretory granules for exocytosis in the actin cortex via interaction with RIM-BP2 and myosin-VIIa. Elife.

[80] Meng YX, Wilson GW, Avery MC, Varden CH, Balczon R (1997). Suppression of the expression of a pancreatic beta-cell form of the kinesin heavy chain by antisense oligonucleotides inhibits insulin secretion from primary cultures of mouse beta-cells. Endocrinology.

[81] Varadi A, Ainscow EK, Allan VJ, Rutter GA (2002). Involvement of conventional kinesin in glucose-stimulated secretory granule movements and exocytosis in clonal pancreatic beta-cells. J Cell Sci.

[82] Cui J, Wang Z, Cheng Q, Lin R, Zhang XM, Leung PS, Copeland NG, Jenkins NA, Yao KM, Huang JD (2011). Targeted inactivation of kinesin-1 in pancreatic beta-cells in vivo leads to insulin secretory deficiency. Diabetes.

[83] Hoboth P, Muller A, Ivanova A, Mziaut H, Dehghany J, Sonmez A, Lachnit M, Meyer-Hermann M, Kalaidzidis Y, Solimena M (2015). Aged insulin granules display reduced microtubule-dependent mobility and are disposed within actin-positive multigranular bodies. Proc Natl Acad Sci USA.

[84] Hirokawa N, Niwa S, Tanaka Y (2010). Molecular motors in neurons: transport mechanisms and roles in brain function, development, and disease. Neuron.

[85] Zhu X, Hu R, Brissova M, Stein RW, Powers AC, Gu G, Kaverina I (2015). Microtubules negatively regulate insulin secretion in pancreatic beta cells. Dev Cell.

[86] Newsholme P, Krause M (2012). Nutritional regulation of insulin secretion: implications for diabetes. Clin Biochem Rev.

[87] Bergsten P (2002). Role of oscillations in membrane potential, cytoplasmic Ca2+, and metabolism for plasma insulin oscillations. Diabetes.

[88] Cerasi E, Luft R (1967). The plasma insulin response to glucose infusion in healthy subjects and in diabetes mellitus. Acta Endocrinol.

[89] Tengholm A, Gylfe E (2009). Oscillatory control of insulin secretion. Mol Cell Endocrinol.

[90] Curry DL, Bennett LL, Grodsky GM (1968). Dynamics of insulin secretion by the perfused rat pancreas. Endocrinology.

[91] Olofsson CS, Göpel SO, Barg S, Galvanovskis J, Ma X, Salehi A, Rorsman P, Eliasson L (2002). Fast insulin secretion reflects exocytosis of docked granules in mouse pancreatic B-cells. Pflüg Arch.

[92] Barg S, Galvanovskis J, Gopel SO, Rorsman P, Eliasson L (2000). Tight coupling between electrical activity and exocytosis in mouse glucagon-secreting alpha-cells. Diabetes.

[93] Bratanova-Tochkova TK, Cheng H, Daniel S, Gunawardana S, Liu YJ, Mulvaney-Musa J, Schermerhorn T, Straub SG, Yajima H, Sharp GWG (2002). Triggering and augmentation mechanisms, granule pools, and biphasic insulin secretion. Diabetes.

[94] Rorsman P, Eliasson L, Renström E, Gromada J, Barg S, Göpel S (2000). The cell physiology of biphasic insulin secretion. Physiology.

[95] Barg S, Eliasson L, Renstrom E, Rorsman P (2002). A subset of 50 secretory granules in close contact with L-type Ca2+ channels accounts for first-phase insulin secretion in mouse-cells. Diabetes.

[96] Pedersen MG, Sherman A (2009). Newcomer insulin secretory granules as a highly calcium-sensitive pool. Proc Natl Acad Sci USA.

[97] Rorsman P, Renström E (2003). Insulin granule dynamics in pancreatic beta cells. Diabetologia.

[98] Greenbaum CJ, Mandrup-Poulsen T, McGee PF, Battelino T, Haastert B, Ludvigsson J, Pozzilli P, Lachin JM, Kolb H, Type 1 Diabetes Trial Net Research, C.P.T.S.G. European (2008). Mixed-meal tolerance test versus glucagon stimulation test for the assessment of beta-cell function in therapeutic trials in type 1 diabetes. Diabetes Care.

[99] Seino S, Shibasaki T, Minami K (2011). Dynamics of insulin secretion and the clinical implications for obesity and diabetes. J Clin Investig.

[100] Straub SG, Shanmugam G, Sharp GWG (2004). Stimulation of insulin release by glucose is associated with an increase in the number of docked granules in the -cells of rat pancreatic islets. Diabetes.

[101] MacDonald PE, Braun M, Galvanovskis J, Rorsman P (2006). Release of small transmitters through kiss-and-run fusion pores in rat pancreatic beta cells. Cell Metab.

[102] Takahashi N, Hatakeyama H, Okado H, Miwa A, Kishimoto T, Kojima T, Abe T, Kasai H (2004). Sequential exocytosis of insulin granules is associated with redistribution of SNAP25. J Cell Biol.

[103] Qin T, Liang T, Zhu D, Kang Y, Xie L, Dolai S, Sugita S, Takahashi N, Ostenson C-G, Banks K, Gaisano HY (2017). Munc18b increases insulin granule fusion, restoring deficient insulin secretion in type-2 diabetes human and goto-kakizaki rat islets with improvement in glucose homeostasis. EBioMedicine.

[104] Zhu D, Koo E, Kwan E, Kang Y, Park S, Xie H, Sugita S, Gaisano HY (2012). Syntaxin-3 regulates newcomer insulin granule exocytosis and compound fusion in pancreatic beta cells. Diabetologia.

[105] Zhu D, Zhang Y, Lam PPL, Dolai S, Liu Y, Cai EP, Choi D, Schroer SA, Kang Y, Allister EM, Qin T, Wheeler MB, Wang C-C, Hong W-J, Woo M, Gaisano HY (2012). Dual role of VAMP8 in regulating insulin exocytosis and islet β cell growth. Cell Metab.

[106] Zhu D, Xie L, Karimian N, Liang T, Kang Y, Huang Y-C, Gaisano HY (2015). Munc18c mediates exocytosis of pre-docked and newcomer insulin granules underlying biphasic glucose stimulated insulin secretion in human pancreatic beta-cells. Mol Metab.

[107] Spurlin BA, Thurmond DC (2006). Syntaxin 4 facilitates biphasic glucose-stimulated insulin secretion from pancreatic β-cells. Mol Endocrinol.

[108] Krus U, King BC, Nagaraj V, Gandasi NR, Sjölander J, Buda P, Garcia-Vaz E, Gomez MF, Ottosson-Laakso E, Storm P, Fex M, Vikman P, Zhang E, Barg S, Blom AM, Renström E (2014). The complement inhibitor CD59 regulates insulin secretion by modulating exocytotic events. Cell Metab.

[109] Michael DJ, Xiong W, Geng X, Drain P, Chow RH (2007). Human insulin vesicle dynamics during pulsatile secretion. Diabetes.

[110] Toonen RF, Kochubey O, de Wit H, Gulyas-Kovacs A, Konijnenburg B, Sørensen JB, Klingauf J, Verhage M (2006). Dissecting docking and tethering of secretory vesicles at the target membrane. EMBO J.

[111] Yasuda T, Shibasaki T, Minami K, Takahashi H, Mizoguchi A, Uriu Y, Numata T, Mori Y, Miyazaki J, Miki T, Seino S (2010). Rim2alpha determines docking and priming states in insulin granule exocytosis. Cell Metab.

[112] Gandasi NR, Barg S (2014). Contact-induced clustering of syntaxin and munc18 docks secretory granules at the exocytosis site. Nat Commun.

[113] Sieber JJ, Willig KI, Kutzner C, Gerding-Reimers C, Harke B, Donnert G, Rammner B, Eggeling C, Hell SW, Grubmuller H, Lang T (2007). Anatomy and dynamics of a supramolecular membrane protein cluster. Science.

[114] Barg S, Knowles MK, Chen X, Midorikawa M, Almers W (2010). Syntaxin clusters assemble reversibly at sites of secretory granules in live cells. Proc Natl Acad Sci USA.

[115] Knowles MK, Barg S, Wan L, Midorikawa M, Chen X, Almers W (2010). Single secretory granules of live cells recruit syntaxin-1 and synaptosomal associated protein 25 (SNAP-25) in large copy numbers. Proc Natl Acad Sci USA.

[116] Bar-On D, Wolter S, van de Linde S, Heilemann M, Nudelman G, Nachliel E, Gutman M, Sauer M, Ashery U (2012). Super-resolution imaging reveals the internal architecture of nano-sized syntaxin clusters. J Biol Chem.

[117] Voets T, Toonen RF, Brian EC, de Wit H, Moser T, Rettig J, Sudhof TC, Neher E, Verhage M (2001). Munc18-1 promotes large dense-core vesicle docking. Neuron.

[118] Ohara-Imaizumi M, Fujiwara T, Nakamichi Y, Okamura T, Akimoto Y, Kawai J, Matsushima S, Kawakami H, Watanabe T, Akagawa K, Nagamatsu S (2007). Imaging analysis reveals mechanistic differences between first- and second-phase insulin exocytosis. J Cell Biol.

[119] Lang T, Bruns D, Wenzel D, Riedel D, Holroyd P, Thiele C, Jahn R (2001). SNAREs are concentrated in cholesterol-dependent clusters that define docking and fusion sites for exocytosis. EMBO J.

[120] Murray DH, Tamm LK (2011). Molecular mechanism of cholesterol- and polyphosphoinositide-mediated syntaxin clustering. Biochemistry.

[121] Balla T (2013). Phosphoinositides: tiny lipids with giant impact on cell regulation. Physiol Rev.

[122] Honigmann A, van den Bogaart G, Iraheta E, Risselada HJ, Milovanovic D, Mueller V, Mullar S, Diederichsen U, Fasshauer D, Grubmuller H, Hell SW, Eggeling C, Kuhnel K, Jahn R (2013). Phosphatidylinositol 4,5-bisphosphate clusters act as molecular beacons for vesicle recruitment. Nat Struct Mol Biol.

[123] Aoyagi K, Sugaya T, Umeda M, Yamamoto S, Terakawa S, Takahashi M (2005). The activation of exocytotic sites by the formation of phosphatidylinositol 4,5-bisphosphate microdomains at syntaxin clusters. J Biol Chem.

[124] James DJ, Khodthong C, Kowalchyk JA, Martin TFJ (2008). Phosphatidylinositol 4,5-bisphosphate regulates SNARE-dependent membrane fusion. J Cell Biol.

[125] Wang J, Richards DA (2012). Segregation of PIP2 and PIP3 into distinct nanoscale regions within the plasma membrane. Biol Open.

[126] Hammond GRV, Schiavo G, Irvine RF (2009). Immunocytochemical techniques reveal multiple, distinct cellular pools of PtdIns4P and PtdIns(4,5)P(2). Biochem J.

[127] Milosevic I, Sorensen JB, Lang T, Krauss M, Nagy G, Haucke V, Jahn R, Neher E (2005). Plasmalemmal phosphatidylinositol-4,5-bisphosphate level regulates the releasable vesicle pool size in chromaffin cells. J Neurosci.

[128] Omar-Hmeadi M, Gandasi NR, Barg S (2018). PtdIns(4,5)P2 is not required for secretory granule docking. Traffic.

[129] Ji C, Fan F, Lou X (2017). Vesicle docking is a key target of local PI(4,5)P2 metabolism in the secretory pathway of INS-1 cells. Cell Rep.

[130] Barg S, Olofsson CS, Schriever-Abeln J, Wendt A, Gebre-Medhin S, Renström E, Rorsman P (2002). Delay between fusion pore opening and peptide release from large dense-core vesicles in neuroendocrine cells. Neuron.

[131] Xu T, Rammner B, Margittai M, Artalejo AR, Neher E, Jahn R (1999). Inhibition of SNARE complex assembly differentially affects kinetic components of exocytosis. Cell.

[132] Zhao Y, Fang Q, Herbst AD, Berberian KN, Almers W, Lindau M (2013). Rapid structural change in synaptosomal-associated protein 25 (SNAP25) precedes the fusion of single vesicles with the plasma membrane in live chromaffin cells. Proc Natl Acad Sci USA.

[133] Lai Y, Choi UB, Leitz J, Rhee HJ, Lee C, Altas B, Zhao M, Pfuetzner RA, Wang AL, Brose N, Rhee J, Brunger AT (2017). Molecular mechanisms of synaptic vesicle priming by Munc13 and Munc18. Neuron.

[134] Gandasi NR, Yin P, Riz M, Chibalina MV, Cortese G, Lund P-E, Matveev V, Rorsman P, Sherman A, Pedersen MG, Barg S (2017). Ca2+ channel clustering with insulin-containing granules is disturbed in type 2 diabetes. J Clin Investig.

[135] Wang S, Li Y, Gong J, Ye S, Yang X, Zhang R, Ma C (2019). Munc18 and Munc13 serve as a functional template to orchestrate neuronal SNARE complex assembly. Nat Commun.

[136] Baker RW, Jeffrey PD, Zick M, Phillips BP, Wickner WT, Hughson FM (2015). A direct role for the Sec1/Munc18-family protein Vps33 as a template for SNARE assembly. Science.

[137] Jockusch WJ, Speidel D, Sigler A, Sørensen JB, Varoqueaux F, Rhee J-S, Brose N (2007). CAPS-1 and CAPS-2 are essential synaptic vesicle priming proteins. Cell.

[138] Abderrahmani A (2004). Complexin I regulates glucose-induced secretion in pancreatic -cells. J Cell Sci.

[139] Bhatnagar S, Oler AT, Rabaglia ME, Stapleton DS, Schueler KL, Truchan NA, Worzella SL, Stoehr JP, Clee SM, Yandell BS, Keller MP, Thurmond DC, Attie AD (2011). Positional cloning of a type 2 diabetes quantitative trait locus; tomosyn-2, a negative regulator of insulin secretion. PLoS Genet.

[140] Dolai S, Xie L, Zhu D, Liang T, Qin T, Xie H, Kang Y, Chapman ER, Gaisano HY (2016). Synaptotagmin-7 functions to replenish insulin granules for exocytosis in human islet β-cells. Diabetes.

[141] Ramalingam L, Lu J, Hudmon A, Thurmond DC (2014). Doc2b serves as a scaffolding platform for concurrent binding of multiple Munc18 isoforms in pancreatic islet β-cells. Biochem J.

[142] Hay JC, Fisette PL, Jenkins GH, Fukami K, Takenawa T, Anderson RA, Martin TFJ (1995). ATP-dependent inositide phosphorylation required for Ca2+-activated secretion. Nature.

[143] Hay JC, Martin TFJ (1993). Phosphatidylinositol transfer protein required for ATP-dependent priming of Ca2+-activated secretion. Nature.

[144] Hay JC, Martin TF (1992). Resolution of regulated secretion into sequential MgATP-dependent and calcium-dependent stages mediated by distinct cytosolic proteins. J Cell Biol.

[145] Xie B, Nguyen PM, Gucek A, Thonig A, Barg S, Idevall-Hagren O (2016). Plasma membrane phosphatidylinositol 4,5-bisphosphate regulates Ca(2+)-influx and insulin secretion from pancreatic beta cells. Cell Chem Biol.

[146] Augustin I, Rosenmund C, Südhof TC, Brose N (1999). Munc13-1 is essential for fusion competence of glutamatergic synaptic vesicles. Nature.

[147] Lemmon MA (2008). Membrane recognition by phospholipid-binding domains. Nat Rev Mol Cell Biol.

[148] Grishanin RN, Kowalchyk JA, Klenchin VA, Ann K, Earles CA, Chapman ER, Gerona RRL, Martin TFJ (2004). CAPS acts at a prefusion step in dense-core vesicle exocytosis as a PIP2 binding protein. Neuron.

[149] Low JT, Zavortink M, Mitchell JM, Gan WJ, Do OH, Schwiening CJ, Gaisano HY, Thorn P (2014). Insulin secretion from beta cells in intact mouse islets is targeted towards the vasculature. Diabetologia.

[150] Ohara-Imaizumi M, Aoyagi K, Ohtsuka T (2019). Role of the active zone protein, ELKS, in insulin secretion from pancreatic β-cells. Mol Metab.

[151] Fu J, Githaka JM, Dai X, Plummer G, Suzuki K, Spigelman AF, Bautista A, Kim R, Greitzer-Antes D, Fox JEM, Gaisano HY, MacDonald PE (2019). A glucose-dependent spatial patterning of exocytosis in human β-cells is disrupted in type 2 diabetes. JCI Insight.

[152] Gan WJ, Do OH, Cottle L, Ma W, Kosobrodova E, Cooper-White J, Bilek M, Thorn P (2018) Local integrin activation in pancreatic cells targets insulin secretion to the vasculature. SSRN Electron J 10.1016/j.celrep.2018.08.035 30208309

[153] Ma W, Chang J, Tong J, Ho U, Yau B, Kebede MA, Thorn P (2020). Arp2/3 nucleates F-actin coating of fusing insulin granules in pancreatic β cells to control insulin secretion. J Cell Sci.

[154] Halban PA, Polonsky KS, Bowden DW, Hawkins MA, Ling C, Mather KJ, Powers AC, Rhodes CJ, Sussel L, Weir GC (2014). Beta-cell failure in type 2 diabetes: postulated mechanisms and prospects for prevention and treatment. Diabetes Care.

[155] Cerasi E, Luft R (1967). “What is inherited - what is added” hypothesis for the pathogenesis of diabetes mellitus. Diabetes.

[156] Gerich JE (2002). Is reduced first-phase insulin release the earliest detectable abnormality in individuals destined to develop type 2 diabetes?. Diabetes.

[157] Lacy PE, Walker MM, Joan Fink C (1972). Perifusion of isolated rat islets in vitro: participation of the microtubular system in the biphasic release of insulin. Diabetes.

[158] Gandasi NR, Yin P, Omar-Hmeadi M, Ottosson Laakso E, Vikman P, Barg S (2018). Glucose-dependent granule docking limits insulin secretion and is decreased in human type 2 diabetes. Cell Metab.

[159] Camunas-Soler J, Dai XQ, Hang Y, Bautista A, Lyon J, Suzuki K, Kim SK, Quake SR, MacDonald PE (2020) Patch-seq links single-cell transcriptomes to human islet dysfunction in diabetes. Cell Metab 10.1016/j.cmet.2020.04.005 PMC7398125 32302527

[160] Fehse F, Trautmann M, Holst JJ, Halseth AE, Nanayakkara N, Nielsen LL, Fineman MS, Kim DD, Nauck MA (2005). Exenatide augments first- and second-phase insulin secretion in response to intravenous glucose in subjects with type 2 diabetes. J Clin Endocrinol Metab.

[161] Hosker JP, Rudenski AS, Burnett MA, Matthews DR, Turner RC (1989). Similar reduction of first- and second-phase B-cell responses at three different glucose levels in type II diabetes and the effect of gliclazide therapy. Metabolism.

[162] Somanath S, Barg S, Marshall C, Silwood CJ, Turner MD (2009). High extracellular glucose inhibits exocytosis through disruption of syntaxin 1A-containing lipid rafts. Biochem Biophys Res Commun.

